# Supplementary material for: A Rapid Prototyping Approach for Multi-Material, Reversibly Sealed Microfluidics
Source: Micromachines (Basel). 2023 Dec 7;14(12):2213. doi: 10.3390/mi14122213 (PMC10745384; doi:10.3390/mi14122213)
Supplement: Supplementary file 1 [file micromachines-14-02213-s001.zip › micromachines-2712588-supplementary.pdf]

Supplementary Information for "A Rapid Prototyping Approach for Multi-material, Reversibly Sealed Microfluidics"

Michael Halwes[1,2], Dr. Melanie Stamp[1,2], Dr. David Collins[1,2]

[1]Department of Biomedical Engineering, University of Melbourne

[2]Graeme Clark Institute for Biomedical Engineering, University of Melbourne

**Parameter**	**6mm Acrylic**	**3mm Acrylic**
**Laser Power (%)**	90	70
**Travel Speed (%)**	.4	.7
**Laser Repetition Rate (Hz)**	5000	1000

Table S1 Laser Cutting Parameters used for the acrylic components of the microfluidic device.
